# The impact of the new cooperative medical scheme on financial burden of tuberculosis patients: evidence from six counties in China

**DOI:** 10.1186/s40249-015-0094-5

**Published:** 2016-01-28

**Authors:** Li Xiang, Yao Pan, Shuangyi Hou, Hongwei Zhang, Kaori D. Sato, Qiang Li, Jing Wang, Shenglan Tang

**Affiliations:** School of Medicine and Health Management, Huazhong University of Science and Technology, Wuhan, China; The Third Affiliated Hospital, Sun Yat-sen University, Guangzhou, China; Hubei Provincial Center for Disease Control and Prevention, Wuhan, China; Shaanxi Provincial Institute for TB Control and Prevention, Xi’an, China; Duke Global Health Institute, Duke University, Durham, NC USA; School of Public Health, Xi’an Jiaotong University, Xi’an, China; Global Health Research Center, Duke Kunshan University, Kunshan, China

**Keywords:** NCMS, Impact, Financial burden, TB

## Abstract

**Background:**

Tuberculosis (TB) patients in China encounter heavy financial burdens throughout the course of their treatment and it is unclear how China’s health insurance systems affect the alleviation of this burden under the integrated approach. This study aimed to measure reimbursement for TB services under the New Cooperative Medical Scheme (NCMS) in rural China and to evaluate changes in catastrophic health expenditure (CHE) caused by the reimbursement policies.

**Methods:**

Reimbursement data were obtained from routine data systems for the NCMS in Yichang (YC) and Hanzhong (HZ). 1884 TB inpatients reimbursed by NCMS from 2010 to 2012 were included. Household surveys were conducted. A total of 494 TB patients under the NCMS were selected in this paper. 12 Focus Group Discussions (FGDs) were held. We measured the impact of the NCMS by counterfactual analysis, which analyzed the financial burden alleviation. Equity was assessed by Concentration Index (CI), and disaggregated by project sites.

**Results:**

TB inpatients were reimbursed with an effective reimbursement rate of 57.3 %. Average out-of-pocket (OOP) payments for outpatient and inpatient services after diagnosis were 1413 yuan and 430 yuan, and 3572 yuan and 3013 yuan in YC and HZ, respectively. The reimbursement level for TB outpatient care after diagnosis was very low due to a limited outpatient quota. TB patients in HZ incurred higher effective reimbursement rates, but the incidence of CHE remained higher. The reduction of CHE incidence after the NCMS showed no difference statistically (P > 0.05). The severity of CHE was alleviated slightly. CIs after reimbursement were all below zero and their absolute values were higher than those before reimbursement.

**Conclusions:**

Low reimbursement for TB patients could lead to heavy financial burden. Poor TB patients incurred high rates of CHE. The NCMS was found to be a protective factor for CHE, but the impact was modest and the equity of CHE did not improve. The NCMS reimbursement policies should be improved in the future to include a more comprehensive coverage of care. Supplemental programs may be necessary to expand coverage for TB care.

**Electronic supplementary material:**

The online version of this article (doi:10.1186/s40249-015-0094-5) contains supplementary material, which is available to authorized users.

## Multilingual abstracts

Please see Additional file 1 for translations of the abstract into the six official working languages of the United Nations.

## Background

China has the second largest burden of tuberculosis (TB) in the world [1], and the “directly observed treatment short course” (DOTS) has been implemented by China’s National TB Program to combat this problem. One smear microscopy and one radiography at first visit, and drugs (6 months for new patients, 8 months if previously treated) are provided free of charge. Despite this, TB patients frequently encounter high costs, often through additional medicines, X-rays and laboratory services, during the course of their treatment [2–4]. Within the current TB control program, two main approaches have been employed in China: the TB dispensary approach and the integrated approach [5]. In the TB dispensary approach, TB dispensaries [usually departments of the Center for Diseases Control (CDC)] are in charge of TB case detection, diagnosis, treatment, and case management. TB suspects and patients should be reported and referred to TB dispensaries to confirm diagnoses and for treatment by health workers in general hospitals. Only severe or complicated TB cases are further treated in general hospitals. Since 2000, TB prevention and treatment services have transformed from TB dispensaries to designated hospitals in what is called the integrated approach. This design allows TB dispensaries to provide public health care and general hospitals to offer integrated care to TB patients without referral. Other hospitals, including township hospitals, are responsible for referring suspects and patients to the designated hospitals. The integrated model has been considered the better policy option for future TB health reform in China [6]. Prior studies have shown that TB poses a heavy financial burden on patients, especially on those in rural areas [7]. A systematic review conducted in low- and middle-income countries has shown that cost as a percentage of income was particularly high among poor people [8]. With the considerable strain that TB places on its population, protecting TB patients from financial risk is a priority for Chinese policymakers.

Social protection mechanisms were highlighted in the post-2015 Global TB Strategy put forward by The World Health Organization. Health insurance has been considered as a promising way to protect people from financial catastrophe and impoverishment [9–11]. As for TB control, in the United States, ongoing efforts to control TB were enhanced by bringing millions of currently uninsured Americans into the health care system [12]. Integrating the national TB control program into health insurance schemes is an effective strategy to address challenges in current China [13]. Lack of health insurance is reported to be the key determinant of insufficient TB control in many ways. According to one prospective cohort study, having health insurance helped to improve latent tuberculous infection treatment completion rates in the United States and Canada [14], while a lack of health insurance was found to be responsible for delay in seeking TB care in China [15].

In rural China, TB patients are reimbursed by the New Cooperative Medical Scheme (NCMS). There is a growing body of literature that evaluates the impacts of the NCMS programs on health care utilization and economic burden [16–20]; however, they mainly focus on the variation of financial burden on the whole population before and after the NCMS, and few target the impacts of different NCMS reimbursement policies on TB patients specifically. A recent study accessed the effective reimbursement rates of the NCMS among TB patients [21], but it compared TB patients who lived in counties where their schemes covered costs within TB dispensaries and those who did not. Nonetheless, the alleviation of financial burden among TB patients during the entire course of treatment under the integrated approach remains unknown. Moreover, more indicators are needed to assess the impacts of the NCMS on financial burden.

TB patients have several distinctive features in their demand for health care. First, from the clinical viewpoint, only a small proportion of TB patients require treatment via inpatient services. More commonly, TB patients need repeated, long-term outpatient visits. Second, under the current health care system in China, primary care physicians practicing in local clinics or public health centers often do not have the capacity to treat TB patients due to resource and educational constraints. Rather, TB patients are treated at county- or city-level hospitals based on the national TB control policies in China. While the NCMS may be beneficial to meet the unmet health care needs of those in rural China [22], under the integrated approach, its impact on alleviating the financial burden on TB patients in rural populations using diversified indexes and whether this impact is consistent with that of the whole population has yet to be unveiled.

This study focused on TB patients in rural areas of central and western China. We measured TB care reimbursement under the NCMS by using data from patient surveys and the NCMS database. We then assessed the impacts of the NCMS on the financial burden of TB patients by counterfactual analysis and discussed whether the NCMS reimbursement policy reached the goal of equity by using the CI.

## Methods

### Study setting

The national TB epidemiology survey in 2010 revealed that there existed significant differences in the prevalence of active pulmonary TB across different regions in China. The prevalence of active pulmonary TB in the western region was about 1.7 times that in the central region, and 2.4 times that in the eastern region. Moreover, the prevalence of active pulmonary TB in rural areas was about 1.6 times that in urban areas. Hence, this paper focuses on TB care reimbursement in rural areas of central and western China.

The China National Health and Family Planning Commission (NHFPC)-Bill & Melinda Gates Foundation TB Project has been widely conducted in Yichang (YC) and Hanzhong (HZ). The local governments were able and willing to collaborate in this study. The study was therefore conducted in these two cities.

YC is located in the more developed area of central China and had a GDP per capita of 73,947 yuan in 2011. By contrast, HZ is a relatively low-income area located in western China, which had a GDP per capita of 16,935 yuan in 2011. YC and HZ adopted the integrated approach. Therefore, TB dispensaries were responsible for TB control planning, case reporting, drug procurement and distribution, defaults tracing and health education. One county-level general hospital was designated for integrated care within one county.

Three counties (one from each category of high, middle and low GDP per capita) were purposively selected in each city. As a result, we chose six counties as our study settings: Yidu (YD), Zhijiang (ZJ) and Wufeng (WF) in YC and Chenggu (CG), Mianxian (MX) and Zhenba (ZB) in HZ.

### Data collection

Data collection employed a combination of both quantitative and qualitative methods. Quantitative data provided intuitional phenomenon, and qualitative data helped to gain a deeper and more complete understanding of the situation. Quantitative studies included TB patient surveys and reimbursement data from the existing routine data systems for the NCMS. Qualitative studies mainly consisted of a number of Focus Group Discussions (FGDs) and policy documents.

### Quantitative study

We adopted probability proportional to size (PPS) sampling to select TB patients. Through this process, we planned to obtain 270 cases per city. Thus, 90 cases per county were needed.

First, townships were randomly selected in each county. We planned to select six townships according to the previous TB case registration list. In ZJ, for example, 370 TB cases were registered in 2012. The sampling interval was 61, and the random number was 29. Accordingly, we listed the sampled patients in a table and the sampled townships were chosen.

Second, 15 TB cases – patients who had completed or stopped treatment during 2012 – were selected in each of the six townships from the TB case registration list. If the sample size of the sampled township was not enough, we supplemented it by choosing from a nearby township. If the sample size was still not enough in this situation, we chose patients who registered in the latter half of 2011 and completed the treatment course.

A standardized questionnaire was designed to conduct patient surveys by face-to-face interviews at local dispensaries or designated hospitals. We collected additional information on personal demographic and socio-economic status (such as education, household income/expenditure), TB related diagnostic and treatment pathway, direct health service expenditures, and on the reimbursements from health insurance. The patient survey was conducted by university students from local cities and provinces. These students received training on interview skills and the contents of the questionnaire. Since this paper focuses on patients with health insurance coverage under the NCMS program, we excluded TB patients with other forms of health insurance schemes. As a result, we had 494 patients in our final sample.

In order to understand the medical costs and reimbursements of TB patients, we collected reimbursement data from January 2010 to December 2012 from the existing NCMS routine data system in each of the six counties. Eligible patients were identified via a TB diagnosis code from the information system. The reimbursement data contained information on the patient’s name, sex, age group, choice of health providers, hospitalization costs, and the reimbursement expenses.

In sum, quantitative data were collected from the TB patient database and the NCMS database. These two databases were complementary. On one hand, due to recall bias, TB patients may have exaggerated their medical expenses to highlight their economic burden and they were only able to remember their out-of-pocket (OOP) payments. The NCMS database, however, provided relatively accurate data about total and reimbursement expenses. On the other hand, medical expenditures beyond the NCMS reimbursement packages may have been excluded by the NCMS managers to report higher reimbursement rates for their counties. By comparing expenditures and reimbursement rates with the patient database, we were able to gain more detailed information. For these reasons, the effective reimbursement rates from the patient database were lower than those from medical expense records.

### Qualitative study

FGDs were held with health care providers from the designated hospitals and TB patients. There were 6 groups each in both YC and HZ. Participants were selected with the help of the local CDCs. One FGD was organized with one stakeholder. The number of participants for each FGD was around 6–8.

The FGDs with the health care providers from the designated hospitals consisted of senior health administrators responsible for public health, heads of TB or infectious disease units, 2–3 chief consultants for TB care, heads of nursing from TB units or wards, and one senior and one junior nurse responsible for TB care. Issues posed included the financing and expenditure of TB care, provision of TB services, TB related health workforce and their income levels, etc.

As for FGDs with patients, inclusion criteria were patients with previous TB treatment experience who could clearly communicate their thoughts. Sex, age group, geographic location of residence, and socio-economic status were considered. Both new and relapsed TB patients were included in the group. Questions regarding their use of and experiences with health care, expenses and reimbursement related to TB care, and their subjective feelings about their financial burden and the usefulness of health insurance were asked.

A total of 12 FGDs were conducted by university faculty members. All FGDs were recorded under the permission of participants. In addition, health insurance policy documents related to the payment and reimbursement of TB treatment as well as provider payment methods were gathered from the study sites.

### Definitions

In our study, total medical expenditure was composed of the actual amount received as reimbursement and any OOP payments. OOP payments were comprised of the expenses below the deductible, the expenses above the deductible which were co-paid by patients and the non-reimbursable amount which were beyond the NCMS benefit packages.

There were two major indicators to empirically measure the reimbursement level: the effective reimbursement rate and the non-reimbursable expenses rate. The effective reimbursement rate was the actual amount received as reimbursement divided by the total medical expenditure [23]. The non-reimbursable expenses rate was the non-reimbursable amount that were beyond the NCMS benefit packages divided by the total medical expenditure.

The definitions mentioned above are consistent with those used by the National Health and Family Planning Commission in China.

It is widely known that there is no commonly accepted criterion for defining CHE [24]. The criteria varies from 10 % of income [25, 26], 10 % of household consumption [27], to 40 % of disposable income [28, 29]. In this paper we regarded OOP health expenditures in excess of 10 % of annual family income as potentially catastrophic.

Analysis of the financial burden alleviation under the NCMS and the extent to which this resulted in catastrophic expenditure before and after receiving the NCMS reimbursement was also accomplished by counterfactual analysis. The state of the households after receiving reimbursement from the NCMS was factual; the state before households received reimbursement (as if they were not covered by the NCMS) was regarded as a counterfactual situation [30].

Two indicators were adapted: incidence and average gap of CHE. We hypothesized that health demand of TB patients would remain the same; then we proceeded to compare the incidence and average gap of CHE due to TB before and after the NCMS reimbursement. The incidence of CHE describes the frequency of households in six counties with health care costs as a share of total expenditure exceeding the chosen threshold in proportion to the sample [Eq. (1)]:

1 $$ \mathrm{C}\mathrm{HEI}={\displaystyle \sum \mathrm{C}\mathrm{I}/\mathrm{N}} $$

CHEI refers to the incidence of CHE. And CI is the catastrophe index. If a TB patient’s OOP payment as a proportion of their annual income is ≥10 %, then CI = 1; otherwise CI = 0. N is the total number of TB patients in the sample.

Average gap of CHE captures the severity of CHE. It describes how much a TB patient’s medical expenditure (as a percentage of their annual income) is in excess of the catastrophic threshold of 10 % of annual income [Eq. (2)]:

2 $$ \mathrm{A}\mathrm{G}={\displaystyle \sum \left(\mathrm{P}-10\%\right)/\mathrm{N}} $$

AG refers to the average gap of CHE. And P is the proportion of OOP cost as a percentage of the annual income of TB patients who incur CHE. N is the total number of TB patients in the sample.

The difference in CHE incidence and average gap before and after reimbursement reflects the impact of the NCMS.

**Fig. 1:**
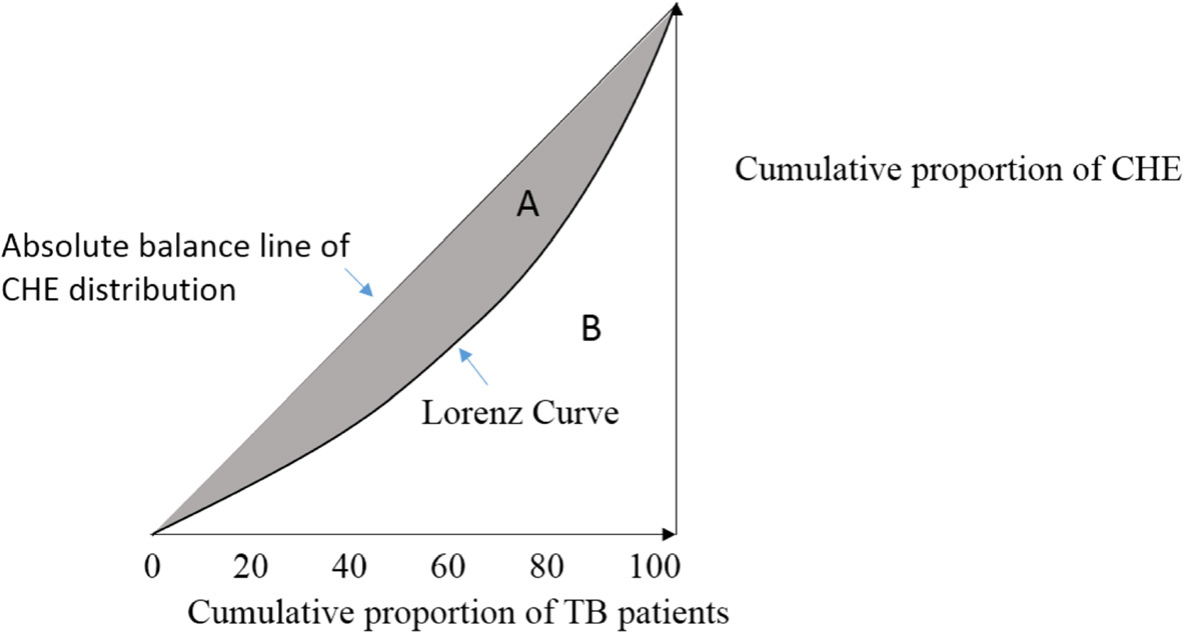
Consideration index of CHE incidence

Equity before and after the NCMS reimbursement was disaggregated by project sites and assessed by CI. Figure 1 presents the CI of CHE incidence rate. The CI is bounded between −1 and 1. For a discrete living standards variable, it can be written as

$$ CI=\frac{2}{N\mu }{\displaystyle {\sum}_{i=1}^N{h}_i{r}_i-1-\frac{1}{N}} $$

Where *h*_*i*_ represents whether an individual suffers from CHE (yes = 1, no = 0), μ is its mean, and ri = i/N is the fractional rank of income, ranging from 0 to 1. For one individual ranked i, r_i = (i-0.5)/N, in which N is the total number of individuals. In this study, the CI was computed by the R programming language.

### Data analysis

We used SPSS 17.0 to conduct the quantitative data analyses. The main analysis focused on the expenditure and reimbursement of TB related services. Chi-square tests were employed to identify differences in effective reimbursement rates between two research cities, and differences in the CHE incidences before and after NCMS reimbursement. *P* < 0.05 was considered to be statistically significant. Total expenses after diagnosis were analyzed to measure TB care reimbursement under the NCMS. In order to assess impact of the NCMS on TB financial burden alleviation, we used expenses throughout the treatment.

The resulting qualitative data were analyzed using thematic framework analysis [31]. Two researchers independently read through all transcripts and listed recruiting viewpoints relevant to questions in the interview guidelines. Codes were developed based on topic guides and the categories that emerged from the transcripts, and were applied to the data to identify themes. All qualitative data were coded, sorted and classified in terms of the analysis framework. Nvivo10 was used to manage the data. Charting was used to identify common or divergent perceptions and explanations were developed. Analysis was carried out in Chinese to keep information consistent and the final results were translated into English.

Documents concerning health insurance policies were also classified and analyzed by region using thematic framework analysis.

### Quality assurance

All the data collection instruments, tools and procedures developed for the study were tested in a pilot exercise in one project city. Following this pilot exercise, a workshop was held to discuss any problems that arose and to identify appropriate modifications as required. A logic check of all the data collected was also undertaken to identify data gaps, inaccuracies, and apparent incongruities and inconsistencies.

The patient surveys were conducted by university students from local cities and provinces. These students were trained on interview skills and the contents of the questionnaire. All transcripts were cross-checked by different persons, allowing the identification and correction of any data entry errors.

The NCMS data from ZJ County were partly excluded due to errors in logic and only data from 2012 were included in the analysis.

### Ethical approval

Ethical permission was obtained from the Institutional Ethics Committee, Chinese Center for Disease Control and Prevention, China.

## Results

### Demographics of TB patients

A total of 494 patients participated in the survey. There was a nearly even distribution within the six selected counties. The average age of the study sample was 54.69 years and about three-fourths of the sample were male. Over half of the participants had a primary school education or lower while 34.8 % completed secondary school and 11.3 % completed high school or higher. At 58.7 %, a majority of the participants were employed,30.8 % were retired, 0.1 % were retired but reemployed, 3.6 % were out of work, and 5.1 % were disabled. And the remaining were students or pre-school children. The average annual household income was 22795 yuan. These 494 patients were divided into three groups by household income. The average annual household incomes were 3552 yuan, 17429 yuan and 53560 yuan in the lower, median and upper quartiles, respectively (Table 1).

**Table 1:** Social-economic characteristics of TB patients (*n* = 494)

Category	Subcategory	Results (% (*n*))
Gender	Female	120 (24.30)
	Male	374 (75.70)
Age	Age (Mean (SD))	54.69 (14.56)
Residence	YD	87 (17.60)
	ZJ	80 (16.20)
	WF	79 (16.00)
	CG	84 (17.00)
	MX	83 (16.80)
	ZB	81 (16.40)
Marital	Married	394 (79.8)
	Unmarried	35 (7.10)
	Divorced	10 (2.00)
	Widowed	55 (11.10)
Education	Primary school and lower	266 (53.80)
	Secondary school or at the same level	172 (34.80)
	High school and higher	56 (11.30)
Employment	Salaried job, farming or self-employment	290 (58.70)
	The retired but reemployment	3 (0.06)
	Unemployment	18 (3.60)
	The retired	152 (30.80)
	Disability	25 (5.10)
	Student/pre-school children	6 (1.20)
Household income	Low (lowest 25)	133 (26.90)
(Missing = 3)	Middle (medium 50)	234 (47.40)
	High (top 25)	124 (25.10)

### Institutional background: The NCMS program in YC and HZ

Since the NCMS is decentralized and administered at the county level [16], we used the county as the unit for our analysis. From health insurance policy documents and FDGs, we gathered that general outpatient service and outpatient service reimbursement for selected catastrophic or chronic diseases existed in the NCMS outpatient reimbursement schemes. General outpatient service only reimburses medical fees for outpatients in primary-level medical and health care institutions, for example, village clinics and township health centers, at a certain proportion [32]. TB patients who must seek treatment in a county-level, TB designated hospital cannot claim reimbursement for general outpatient care. In 2009, the NCMS covered catastrophic or chronic outpatient costs in two-thirds of counties in China [33]. Outpatient service reimbursement for selected catastrophic or chronic diseases compensates large outpatient expenditures by establishing the catastrophic or chronic diseases pooling fund with separate deductibles and reimbursement caps. YC and HZ also developed different payment arrangements for outpatient care for specific catastrophic or chronic disease conditions, including TB. TB outpatient services can be reimbursed from pooled funds. Table 2 presents the reimbursement rules for TB outpatients under the NCMS by area.

**Table 2:** Reimbursement by the NCMS for TB outpatient care in 2012

	Reimbursement rate or quota	Ceiling
YD	Quota subsidy 200 yuan/year	
ZJ	Quota subsidy 45 yuan/month	540 yuan/year
WF	70 % of total annual expenditure	2000 yuan/year
CG	Quota subsidy 800 yuan/year	
MX	80 % of total annual expenditure	1800 yuan/year
ZB	Quota subsidy	New patient ,2000 yuan/year;
		Relapse patient, 2500 yuan/year

Data resource: Author’s data are collected from reimbursement policies of the NCMS offices

Reimbursement for TB inpatient services follows similar trends as other inpatient services in all the NCMS schemes. Inpatient deductibles are generally higher if patients receive treatment at higher level hospitals [34]; hence, the reimbursement rates decrease as the patients seek care at higher level hospitals. Table 3 shows the deductible and reimbursement rates, which indicates that the ceiling level for expense reimbursement ranges from RMB 100,000 to 150,000 yuan.

**Table 3:** Reimbursement by the NCMS for TB inpatient care in 2012

County	Deductible (Yuan)			Nominal reimbursement rate (%)		
	Township health centers	County hospitals	Municipal hospital	Township health centers	County hospitals	Municipal hospital
YD	100	500	500	85	65	50–65
ZJ	100	300	300–500	85	75	50–65
WF	50	200	500	80	70	55–65
CG	100–150	300–450	400–600	85–90	70–75	60–70
MX	100–150	300–550	350–550	85–90	75–80	60–70
ZB	100–150	300–550	400–550	80–90	70–80	60–70

Data resource: Author’s data are collected from reimbursement policies of the NCMS offices

### TB care reimbursement under the NCMS

**Fig. 2:**
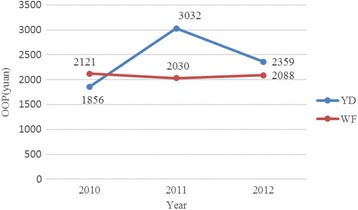
OOP in YD and WF by year

**Fig. 3:**
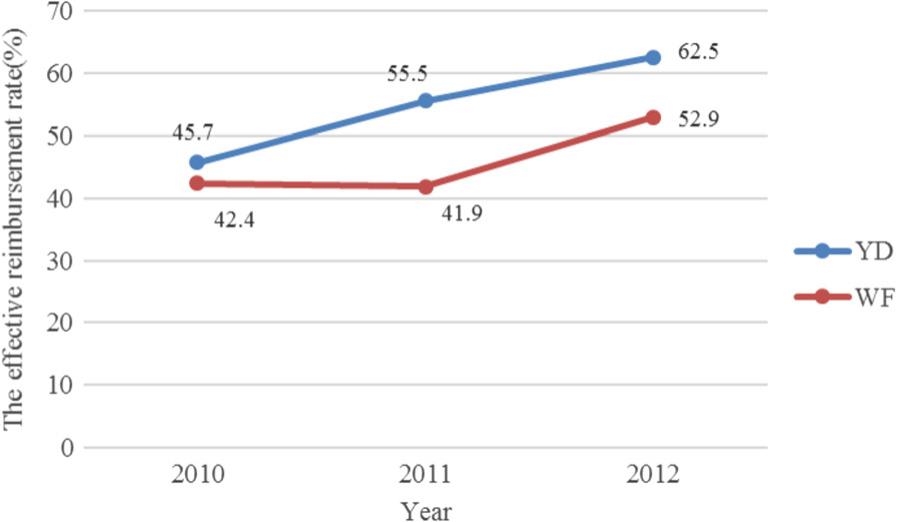
The effective reimbursement rate in YD and WF by year

According to the NCMS database 1884 TB inpatients were reimbursed from 2010 to 2012 in YC and HZ, as shown in Table 4. The rates of non-reimbursable expenses accounted for 8.9 and 9.0 % of total inpatient expenses in YC and HZ, respectively. In addition, we divided OOP payments and the effective reimbursement rates by year. Given the reliability of data, only data in YD and WF were included (Figs. 2 and 3). These figures show the increase in effective reimbursement rates by year, while OOP payments showed no obvious decline.

**Table 4:** TB inpatient care reimbursement under the NCMS in YC and HZ from 2010 to 2012

County	Number	Total inpatient expenses (Yuan)		OOP (Yuan)		Rate of non- reimbursable expenses (%)	The effective reimbursement rate (%)
		Mean	Median	Mean	Median		
YD	430	4522	3106	2397	1513	7.8	47.0
ZJ	242	6372	4734	3048	1972	13.3	52.2
WF	329	4818	3148	2078	1195	5.8	56.9
Total	1001	5067	3602	2450	1609	8.9	51.6
CG	231	3878	3276	1730	1437	16.9	55.4
MX	233	4767	3907	1651	1376	13.7	65.4
ZB	419	4595	4131	1462	1281	2.7	68.2
Total	883	4453	3862	1582	1358	9.0	64.5

Data resource: Author’s data are collected from the NCMS database

Note: In YC, the NCMS data for three years are comprehensive with one exception of ZJ where only data in 2012 are included in the analysis. Given quality of data, data in MX and ZB in 2010 are excluded in the analysis

Table 5 shows TB outpatient and inpatient care reimbursement under the NCMS in YC and HZ using the TB patient database. The effective reimbursement rate of TB inpatient care after diagnosis accounted for 51.9 and 45.9 % in YC and HZ, respectively. There was no statistically significant difference in the effective reimbursement rate for inpatient services between YC and HZ (*P* > 0.05).

**Table 5:** TB outpatient and inpatient care reimbursement after diagnosis under the NCMS in YC and HZ from 2010 to 2012

Area	Outpatient			Inpatient		
	Total Expenses (Yuan)	OOP (Yuan)	ERR (%)	Total Expenses (Yuan)	OOP (Yuan)	ERR (%)
YD	1379	1307	5.2	9086	3947	56.6
ZJ	1942	1903	2.0	14732	7535	48.9
WF	1032	938	9.1	13247	6143	53.6
Total	1490	1413	5.2	13562	6530	51.9
CG	486	451	7.2	11425	7636	33.2
MX	751	584	22.2	7410	3766	49.2
ZB	1189	276	76.8	6542	2619	60.0
Total	832	430	48.3	8234	4454	45.9

Data resource: Author’s data are collected from TB patient database

Note: ERR refers to “the effective reimbursement rate”

From the FGDs with doctors in designated hospitals, we found that TB inpatients were confronted with repeated treatment. We then analyzed service utilization of TB patients who were interviewed and found that 44.13 % of these patients received inpatient care. A majority of TB inpatients (92.13 %) were hospitalized in county or higher level hospitals for initial treatment after diagnosis. On the other hand, 17.13 % sought medical care in municipal hospitals, resulting in lower reimbursement rates. Patients’ repeated treatment and high hospitalization rates were caused by individual and health facility factors, according to the FGDs.

*“TB patients receive low outpatient reimbursement rates. They prefer to be hospitalized, because they can claim higher reimbursement rates, and it is more comfortable and effective from their point of view. Some patients think, ‘At least I can get a general check-up.’”(Doctors in hospitals, FGDs)*

*“(The subsidy for doctors is insufficient), we make money by ourselves.” (TB doctors, FGDs)*

*“They (Doctors) have profit-seeking behavior.” (TB patients, FGDs)*

Total outpatient expenses were near 1500 yuan after diagnosis in YC, and it was about 800 yuan in HZ (see Table 5). Outpatient expenses were high in certain counties, particularly in ZJ. The reimbursement level for TB outpatient care after diagnosis was very low. The effective reimbursement rate of TB outpatient care accounted for 5.2 and 48.3 % in YC and HZ, respectively. There were significant differences (P < 0.05) in effective reimbursement rate for outpatient treatment between YC and HZ. TB patients reflected that the reimbursement for TB outpatient care was limited effect.

As mentioned above, the NCMS covered a package of catastrophic or chronic disease-related services and TB was included in YC and HZ. But the reimbursement was based on a quota or was limited to a certain insured amount. TB outpatients had to pay all expenses above the quota out of pocket. In YD and ZJ, the quota levels for TB outpatient care were significantly lower than the corresponding outpatient expenses and that in HZ, resulting in lower reimbursement levels compared to counties in HZ. Moreover, reimbursement packages for TB outpatient care were less comprehensive than those for TB inpatient care. Outpatient reimbursement packages in most counties excluded many services covered by inpatient reimbursement packages, including CT scans, which further reduced the proportion of effective outpatient reimbursement.

*“The reimbursement for outpatient care was low, and it had no effect. I only got reimbursed for 200 yuan. ” (TB patients, FGDs)*

### Impact of the NCMS on TB Financial Burden Alleviation

According to the Pearson’s chi square tests, there was no statistically significant difference in the CHE incidence before and after the NCMS reimbursement (*P* > 0.05), with the exception of MX (*P* = 0.035). Most catastrophic patients remained catastrophic; however, the average gap of CHE was reduced after TB patients were reimbursed from the NCMS, indicating a slight decline in the severity of CHE. The variation in average gap (Table 6) suggests that the reimbursement policies of the NCMS were effective in reducing CHE, but the impact was modest.

**Table 6:** Incidence and average gap of CHE, and concentration index of CHE incidence rate before and after the NCMS reimbursement by county

Counties	Incidence			Average gap			Concentration index		
	Pre-	Post-	Reduction	Pre-	Post-	Reduction	Pre-	Post-	Reduction
YD	52.0	48.0	4.0	20.9	15.3	5.6	−0.34	−0.30	−0.04
ZJ	85.7	85.7	0.0	76.8	55.4	21.4	−0.12	−0.12	0.00
WF	70.0	56.7	13.3	40.0	18.6	21.4	−0.16	−0.32	0.16
CG	73.4	68.8	4.6	131.2	77.8	53.4	−0.16	−0.20	0.04
MX	87.3	70.9	16.4	199.1	108.5	90.7	−0.07	−0.24	0.17
ZB	80.3	72.1	8.2	198.3	126.2	72.1	−0.15	−0.17	0.02

Note: “Reduction” refers to reduction by the NCMS. It indicates the difference before and after the NCMS reimbursement

The CI, our measure of equity, was negative for the NCMS reimbursement in all counties, indicating that in all cases CHE incidence rate was pro-poor. Poorer patients were particularly vulnerable, with high rates of catastrophic spending. But the absolute values of the CI for CHE incidence rate were all below 0.4, suggesting that equity was relatively reasonable. After the NCMS reimbursement, the harsh economic effects of TB on poorer populations were even worse, with the exception of YD.

## Discussion

A review of studies that measured the economic costs of diseases, including TB, showed that low coverage and user charges led to high costs [35]. Pan HQ et al. interviewed 316 patients diagnosed from January 2010 to May 2011 who had already completed their anti-TB treatment in a cross-sectional survey, and found that the average per capita total OOP payment was 3024.0 yuan. They pointed out that the economic burden on patients was still heavy [36]. Long Q et al. found that low income patients paid a total of US$ 149 to 724 (RMB 1241 to 5228) for medical costs for a treatment course [37]. In 2011, the participation rate of NCMS was over 97 % [38]. A majority of TB patients in rural China can be covered by the NCMS, but as a government-subsidized rural health insurance, it mainly focuses on helping cover catastrophic medical expenses [39]. Its impact on the alleviation of the economic burden of TB remains to be seen. By accessing the effective reimbursement rates of the NCMS among TB patients, Wei X et al. found that the NCMS has not relieved the financial burden of TB-related medical costs; however, they focused on the TB dispensary approach.

Overall, our study revealed a mixed picture in terms of TB care reimbursement, and how the NCMS has influenced the level of financial burden and equity of TB patients under the integrated approach. More specifically, the study yielded four major findings.

We found that TB patients paid out of pocket for ancillary services, which were not covered by the NCMS [40], and revealed that TB outpatient care reimbursement remained inadequate. The quota subsidy for TB outpatient care led to inadequate outpatient reimbursement. Small TB outpatient expenditures may seem to be insignificant, but the annual cumulative amount maybe catastrophic. Yip and her colleagues pointed out that the NCMS does not address a major cause of medical impoverishment: expensive outpatient services for chronic conditions [41]. Disparities between inpatient and outpatient reimbursement policies possibly led to the high hospitalization rates for TB patients. The hospitalization costs were significantly higher, resulting in financial burden for TB patients. Thus, prevention of hospitalizations are needed [39].

We also found that effective reimbursement rates were far lower than the nominal reimbursement rates for inpatient care. There were three possible explanations for low reimbursement rates for TB inpatient care. First, TB patients had to be hospitalized at medical institutions at the county level or above. In our study, 92.13 % of patients were treated at county hospitals or higher, but reimbursement rules were typically less generous for TB patients hospitalized in higher level facilities. Large deductibles (ranging from 200 yuan to 500 yuan in YC and 300 yuan to 600 yuan in HZ) and high co-payments (ranging from 30 to 50 % in YC and 20 to 40 % in HZ) reduced the proportion of effective reimbursement. As a result, patients had to pay more OOP. Second, TB reimbursement packages were not comprehensive and certain expenses were excluded, including liver protection drugs. Take the medicine reimbursement list of Hubei province as an example – only eleven anti-TB items were included in the NCMS, including streptomycin, isoniazid, rifampicin, ethambutol, sodium aminosalicylate, pyrazinamide, rifapentine and rifamycin. Third, doctors in TB designated hospitals had strong financial incentives for excessive treatment under fee-for-service payment systems, including the use of second-line anti-TB drugs which were expensive and not included in reimbursement schemes. For example, the rate of non-reimbursable expenses accounted for 10.07 % of total medical expenditure in two cities.

The effective reimbursement rate for TB inpatient care has increased over the years, while the OOP payments have not. It appears that most of the reimbursement from the NCMS was offset by the growth of medical expenses [33], which was consistent with the analysis by Wagstaff et al. who found that the NCMS did not reduce OOP payments per outpatient visit or inpatient spell [22]. Financial burden experienced by TB patients can cause delays, low completion rates, poor adherence and drug resistance, as well as CHE and impoverishment [42, 43].

Reimbursement policy design for TB care led to differences in effective reimbursement rates between regions. Higher nominal reimbursement rates and ceilings for TB inpatient and outpatient care were offered in HZ when compared with YC, as shown in Tables 2 and 3. TB patients in HZ encountered lower medical expenditures and higher effective reimbursement rates, but the incidence and severity of CHE remained higher when compared with YC. This indicates that lower-income TB patients in resource-poor settings bear a heavier financial burden, which is in line with other studies [44, 45].

In YC, the reimbursement level for TB outpatient and inpatient care in ZJ was significantly lower than that in YD and WF, and medical expenses for outpatient and inpatient treatment were much higher. Thus, TB patients in ZJ incurred the highest OOP payments. Though the economic level of ZJ ranked in the middle of the three sample counties in YC, the incidence and severity of CHE remained the most serious. It shows that the main factors associated with the incidence and severity of CHE are medical expenditure and reimbursement level without considering other confounding factors.

TB patients paid a high proportion of medical care expenditure through OOP payments after the NCMS reimbursement. Poor TB patients incurred high rates of catastrophic spending due to inadequate reimbursement, in line with other studies which have found that the occurrence and intensity of CHE was greater among poor inpatients [10, 46, 47]; however, equity remained relatively reasonable. A possible explanation may be that the economically disadvantaged group constrained their essential health demand, which in turn is another avenue for further research.

We found the NCMS to be a protective factor in reducing the severity of CHE in particular, which was consistent with other studies [10, 18]. But the NCMS did not reduce the CHE incidence, with the exception of MX, due to inadequate outpatient and inpatient reimbursement levels, a finding that was in line with previous studies [44], and only the severity of CHE was relieved.

The equity of CHE incidence has not improved after the implementation of the NCMS. The effective reimbursement rate remains low under the NCMS, and since the program targets reimbursement for overall beneficiaries, equity improvement for TB patients is negatively affected.

Establishing the National Medical Financial Assistance Scheme (MFA) for the poor or disadvantaged may be effective in equity improvement. In 2003, the MFA was implemented in rural areas to assist, among others, poor and near-poor households facing high health care expenses [22]. Although the MFA was established across these six counties, the vast majority of TB patients could not meet the requirements to obtain the assistance services due to the strict admittance conditions.

Our study has its own limitations. First, since it was based on counterfactual analysis, the level of medical expenditures was assumed to be unchanged before and after NCMS reimbursement, while behavioral responses of patients and providers under reimbursement might have changed, which would have led to an increase in health expenditures. Thus, the impact of the NCMS on the alleviation of economic burden might be overestimated. This also reveals the fact that the NCMS has a modest impact on reducing the financial burden experienced by TB patients. Second, YC and HZ are two cities with different social and economic levels. Different reimbursement policies were employed. Thus, in order to eliminate confounding factors, comparisons were only made within each city.

## Conclusion

This study examined the impact of the NCMS on TB care reimbursement and its effect on the financial burden of TB patients. A number of conclusions can be drawn from our findings. First, low reimbursement for TB outpatients and inpatients due to low reimbursement quotas led to heavy financial burdens on TB patients. Second, the effective reimbursement rates were lower than the reported nominal reimbursement rates for inpatient care. We also discovered a discrepancy between the lower effective reimbursement rates from the patient database and the higher rates from medical expense records. Third, poorer TB patients incurred higher rates of CHE, leaving lower income patients in a more vulnerable position despite the implementation of the NCMS. Fourth, the NCMS was found to be a protective factor for CHE, but the impact was modest. Furthermore, the equity of CHE did not improve after the NCMS reimbursement.

Our findings are valuable for policymakers to develop more effective interventions to relieve the financial burden of TB patients enrolling in the NCMS. The major policy implications from our study are twofold. First, increasing the reimbursement level for TB outpatients and reducing the variations in the reimbursement level across counties are two important avenues to alleviate the financial burden for TB patients and to increase the equity of health insurance systems. Second, since TB patients need to spend significant amounts of money on non-medical costs as well as on non-reimbursable medical costs during the treatment process, relying on a single policy tool such as health insurance coverage may not completely solve the problem of high financial burdens. A supplemental policy tool such as establishing an MFA program may be necessary to improve equity.

## Additional file

Additional file 1:
**Multilingual abstracts in the six official working languages of the United Nations.** (PDF 398 kb)

## Abbreviations

CHE: catastrophic health expenditure
CI: concentration index
CG: Chenggu County
FGDs: focus group discussions
CDC: Center of Disease Control and Prevention
DOTS: directly observed treatment short course
HZ: Hanzhong City
MX: Mianxian County
MFA: the National Medical Financial Assistance Scheme
NCMS: New Cooperative Medical Scheme
OOP: out-of-pocket
TB: Tuberculosis
WF: Wufeng County
YC: Yichang City
YD: Yidu County
ZJ: Zhijiang County
ZB: Zhenba County
