# Supplementary material for: The impact of the new cooperative medical scheme on financial burden of tuberculosis patients: evidence from six counties in China
Source: Infect Dis Poverty. 2016 Jan 28;5:8. doi: 10.1186/s40249-015-0094-5 (PMC4730613; doi:10.1186/s40249-015-0094-5)
Supplement: Additional file 1: — Multilingual abstracts in the six official working languages of the United Nations. (PDF 398 kb) [file 40249_2015_94_MOESM1_ESM.pdf]

Translation of the abstract into the six official working languages of the United Nations

تأثير النظام الطبي التعاوني الجديد على الأعباء المادية لمريض السل الرئوي بمدن الصين الستة

لي زيانج، ياو بان، شانجي هو، كواري دي ساتو، كيانج لي، جينج وانج، شنجلان تانج

ملخص:

مقدمة: يواجه مريض السل الرئوي بالصين أعباء مادية طوال فترة علاجه ويعد تأثير نظام التأمين الصحي لتخفيف العبء غير واضح خلال النهج المتكامل. تهدف هذه الدراسة إلى قياس التحصيل عن خدمات السل الرئوي من خلال النظام الطبي التعاوني الجديد داخل الريف الصيني ولتقييم التغييرات بتكاليف العلاج الكارثية والتي تسببت بها السياسات المالية.

منهج البحث: جمعت بيانات التكاليف من نظم بيانات روتينية للنظام الطبي التعاوني الجديد لكل من يشانج وهانزونج . اشتملت الدراسة على 1884 مريض سل رئوي للنظام الطبي التعاوني الجديد من عام 2010 إلى 2012. أجريت معاينات منزلية. اختير 494 مريض بالسل الرئوي الخاضعين للنظام الطبي التعاوني الجديد للمشاركة بالبحث. أقيمت 12 مجموعة تركيز نقاشية. قاس البحث تأثير النظام الطبي التعاوني الجديد على تحليل الواقع المضاد والذي تم فيه تحليل تخفيف العبء المادي. قيم البحث المساواة من خلال معامل التركيز ومقسم طبقًا للمواقع.

النتائج: تحمل مرضى السل الرئوي معدل مؤثر بقيمة 57.3% . يقدر متوسط قيمة السداد خارج الجيب لخدمات مرضى العيادات الخارجية والمرضى النزلاء بالمستشفى ب1413 ايوان و430 أيوان و3572 ايوان و3014 ايوان بكل من يشانج وهانزونج على التوالي. مستوى التكاليف لمرضى السل الرئوي بالعيادات الخارجية بعد التشخيص منخفض ولكن استمر نمط حدوث التكاليف العلاجية الكارثية عالي. لم يظهر فرق باتخفاض معدل التكاليف العلاجية الكارثية بعد تطبيق النظام الطبي التعاوني الجديد من الناحية التحليلية (المعدل أكبر من 0.05). خففت حدة التكاليف العلاجية العالية بصورة بسيطة. قيمة معامل التركيز بعد التكاليف اقل من صفر والقيم المطلقة أعلى من تلك قبل التكاليف.

الخلاصة: قد يؤدي انخفاض التكاليف لمرضى السل الرئوي إلى معامل حماية عالي. يتحمل مرضى السل الرئوي الفقراء معدلات عالية من تكاليف العلاج الكارثية. أظهرت النتائج أن النظام الطبي التعاوني الجديد عامل وقائي لتكاليف العلاج الكارثية ولكن التأثير بسيط ولكن لم تتحسن التكاليف العلاجية الكارثية. يجب تعديل سياسات تكاليف النظام الطبي التعاوني الجديدفي المستقبل لتضمين قرارات شاملة للتغطية. يجب استخدام برامج إضافية لتوسيع التغطية شبكة العناية بمرضى السل الرئوي.

Translated from English version into Arabic by laila mostafa, through

TRANSLATORS
WITHOUT BORDERS

新型农村合作医疗对结核病患者疾病经济负担的影响——基于中国六个县的实证研究

项莉，潘瑶，侯双翼，张宏伟，Kaori D. Sato，李强，王静，汤胜蓝

摘要：

背景：在中国，结核病患者全程治疗的疾病经济负担十分沉重。然而，在医防合作的模式下，中国基本医疗保障制度对结核病患者疾病经济负担的影响尚不明确。本研究旨在探究在中国农村地区，新型农村合作医疗（以下简称“新农合”）对结核病的补偿情况，并评估不同保障制度对灾难性卫生支出发生情况所产生的影响。

方法：本研究从宜昌、汉中两市的新农合信息系统中获取补偿数据，2010-2012 年共计 1884 名结核病患者获得新农合的补偿。通过入户调查，共计 494 名参与新农合的结核病患者被纳入研究。共进行了 12 组焦点小组访谈。本研究通过反事实分析法分析新农合患者疾病经济负担的效果。运用集中指数，以市为单位，探究保障制度的公平性。

结果： 结核病住院患者的实际补偿比为 57.3%。门诊和确诊后住院的平均自付费用宜昌分别为 1413 元和 3572 元，汉中分别为 430 元和 3013 元。由于门诊限额补偿，结核病患者确诊后门诊补偿水平非常低。汉中的结核患者实际补偿比较高，然而灾难性卫生支出发生率依然很高。新农合补偿后，灾难性卫生支出发生率的减少并无统计学差异（P＜00.05），灾难性卫生支出的严重程度有所减轻。新农合补偿后，集中指数均小于零，且他们的绝对值均高于新农合补偿前。
结论： 较低的补偿水平将导致较为沉重的疾病经济负担。贫困的结核病患者灾难性卫生支出发生率较高。新农合对于防范灾难性卫生支出有积极作用，但是作用有限，且灾难性卫生支出发生的公平性并没有改善。新农合应该完善补偿政策，扩大补偿范围。补充的制度对于扩大结核病的受益面很有必要。

Translated from English version into Chinese by Xiang Li

L'impact du Nouveau régime médical coopératif sur le fardeau financier des patients tuberculeux : données de six comtés de la Chine

Li Xiang, Yao Pan, Shuangyi Hou, Hongwei Zhang, Kaori D. Sato, Qiang Li, Jing Wang, Shenglan Tang

Résumé
Contexte : Les patients atteints de tuberculose en Chine portent un lourd fardeau financier tout au long de leur traitement, et l'on ne connaît pas l'incidence des régimes d'assurance chinois sur l'allègement de ce fardeau en vertu de l'approche intégrée. Cette étude visait à mesurer le remboursement des soins associés à la tuberculose sous le Nouveau régime médical coopératif en Chine rurale, et à évaluer les changements dans les dépenses astronomiques en soins de santé causés par les politiques de remboursement.
Méthodologie : Les données sur le remboursement proviennent des systèmes de compilation ordinaire de renseignements du Nouveau régime médical coopératif des comtés de Yichang (YC) et de Hanzhong (HZ). Les données sur 1 884 patients hospitalisés pour la tuberculose qui ont été remboursés par le Nouveau régime médical coopératif de 2010 à 2012 ont été incluses. Des enquêtes auprès des ménages ont été menées. 494 tuberculeux assujettis au Nouveau régime médical coopératif ont été sélectionnés pour les besoins de cette étude. 12 discussions de groupe ont eu lieu. Nous avons mesuré l'impact du Nouveau régime médical coopératif par une analyse hypothétique de l'allègement du fardeau financier. L'équité a été évaluée au moyen de l'indice de concentration et ventilée par site de projet.
Résultats : Les patients tuberculeux hospitalisés ont été remboursés à un taux effectif de 57,3 %. À la suite du diagnostic, la moyenne des dépenses remboursables pour les patients externes et les patients hospitalisés était de 1 413 yuans et 430 yuans, et de 3 572 yuans et 3 013 yuans à Yichang et Hanzhong respectivement. À la suite du diagnostic, le niveau de remboursement pour les patients tuberculeux en consultation externe était très faible en raison d'un quota limitant les consultations externes. Les patients atteints de tuberculose de Hanzhong ont bénéficié d'un taux de remboursement effectif plus élevé, mais l'incidence des dépenses astronomiques en soins de santé est demeurée plus élevée. La réduction de l'incidence des dépenses astronomiques en soins de santé après l'adoption du Nouveau régime médical coopératif n'a donné lieu à aucune différence statistiquement mesurable (P > 0,05). L'incidence des dépenses astronomiques en soins de santé a été légèrement atténuée. Les indices de concentration après remboursement étaient tous inférieurs à zéro et leurs valeurs absolues étaient plus élevées que celles d'avant le remboursement.
Conclusions : Le faible taux de remboursement pourrait entraîner une lourde charge financière pour les patients tuberculeux. Les patients tuberculeux pauvres doivent assumer des dépenses astronomiques en soins de santé. Le

Nouveau régime médical coopératif s'est avéré un facteur de protection à l'égard des dépenses astronomiques en soins de santé, mais son impact a été modeste, et l'équité des dépenses astronomiques en soins de santé ne s'est pas améliorée. Les politiques de remboursement du Nouveau régime médical coopératif doivent être améliorées afin d'offrir une couverture plus complète des soins. Des programmes complémentaires pourraient être nécessaires afin d'élargir la couverture pour les soins associés à la tuberculose.

Translated from English version into French by Jean Heon, through

TRANSLATORS WITHOUT BORDERS

Влияние Новой схемы кооперативного страхование на облегчение финансовых трудностей туберкулёзных больных: данные по шести округам Китая

Ли Сян (Li Xiang), Яо Пань (Yao Pan), Шуани Хоу (Shuangyi Hou), Хунвэй Чжан (Hongwei Zhang), Каори Д. Сато (Kaori D. Sato), Цян Ли (Qiang Li), Цзин Ван (Jing Wang), Шэнлань Тан (Shenglan Tang)

Аннотация

Краткое описание: Больные туберкулёзом (ТБ) в Китае сталкиваются с серьёзными финансовыми трудностями при прохождении курса лечения, и неизвестно, как системы медицинского страхования в стране помогают облегчить эти трудности при интегрированном подходе к проблеме. Данное исследование было направлено на оценку сумм выплат на лечение ТБ по Новой схеме кооперативного страхования (NCMS) в сельских районах Китая и того, как положения о компенсации затрат повлияли на катастрофически высокие медицинские расходы (CHE).

Методы: Данные о компенсациях были получены из автоматизированных систем NCMS в городах Ичан (Yichang) и Ханьчжон (Hanzhong). В эти данные входили сведения о 1884 стационарных больных ТБ, получивших выплаты от NCMS с 2010 по 2012 год. Были выполнены обследования домашних хозяйств. Для этой научной работы были отобраны 494 больных ТБ, пользовавшихся NCMS. Были проведены 12 дискуссий фокус-групп. Для анализа результатов NCMS мы применили контрфактивный подход. Для оценки неравенства был использован индекс концентрации с разбивкой по местам проведения проекта.

Результаты: Фактический показатель возмещения расходов для стационарных больных ТБ составил 57,3%. В среднем собственные расходы пациентов на амбулаторное и стационарное лечение после диагностирования были 1413 юаня и 430 юаня, и в Ичане и Ханьчжоне 3572 юаня и 3013 юаня соответственно. Процент компенсации затрат на амбулаторное лечение после диагностирования был очень низким вследствие ограниченной квоты на амбулаторное лечение. Больные ТБ в Ханьчжоне получали более высокий процент компенсаций, однако доля расходов CHE оставалась высокой. Уменьшение доли расходов CHE после введения NCMS статистически ничего не изменило ($P > 0,05$). Степень серьёзности CHE слегка снизилась. Все индексы концентрации после выплаты компенсаций были ниже нуля, и их абсолютные величины повысились по сравнению со значениями до компенсаций.

Выводы: Низкий уровень компенсаций для больных ТБ может приводить к серьёзным финансовым трудностям. Малоимущие больные ТБ сталкиваются с высокой долей расходов CHE. Было установлено, что схема NCMS является защитным фактором от CHE, но её воздействие оказалось незначительным, и

доля CHE не уменьшилась. В будущем положения NCMS о компенсации затрат следует улучшить, чтобы включить более всестороннее покрытие услуг. Возможно необходимо введение дополнительных программ для расширения покрытия расходов на лечение ТБ.

Translated from English version into Russian by Natalia Potashnik, through

TRANSLATORS WITHOUT BORDERS

El impacto del Nuevo programa médico cooperativo sobre la carga de los pacientes con tuberculosos: evidencia de seis condados en China

Li Xiang, Yao Pan, Shuangyi Hou, Hongwei Zhang, Kaori D. Sato, Qiang Li, Jing Wang, Shenglan Tang

Resumen

Antecedentes: Los pacientes con tuberculosis (TB) en China se enfrentan a grandes cargas financieras durante todo el trascurso de su tratamiento y no está claro de qué manera los sistemas de seguro de salud chinos permiten aliviar esta carga dentro del enfoque integrado. En este estudio, el objetivo era medir el reembolso de los servicios para TB en el marco del Nuevo programa médico cooperativo (*New Cooperative Medical Scheme*, NCMS) en la China rural y evaluar los cambios en los gastos catastróficos en salud (*catastrophic health expenditure*, CHE) que causan las políticas de reembolso.

Métodos: Se obtuvieron los datos sobre reembolsos de los sistemas de datos de rutina del NCMS en Yichang (YC) y Hanzhong (HZ). Se incluyeron 1 884 pacientes hospitalizados con TB que recibieron reembolsos del NCMS entre 2010 y 2012. Se realizaron encuestas en los hogares. Se seleccionaron 494 pacientes con TB del programa NCMS para este artículo. Hubo 12 debates de grupos focales (Focus Group Discussions, FGD). Medimos el impacto del NCMS a través de análisis contrafáctico, que analizó el alivio de la carga financiera. Para evaluar la equidad, que se dividió según los centros del proyecto, se usó el índice de concentración (*Concentration Index*, CI).

Resultados: Los pacientes hospitalizados recibieron reembolsos con una tasa de reembolso efectivo del 57,3 %. Los pagos promedio de bolsillo (*out-of-pocket*, OOP) para servicios de pacientes hospitalizados y ambulatorios después del diagnóstico fueron de 1 413 yuanes y 430 yuanes, y de 3 572 yuanes y 3 013 yuanes en YC y HZ, respectivamente. El nivel de reembolsos para la atención ambulatoria de la TB después del diagnóstico fue muy bajo debido a una cuota limitada de pacientes ambulatorios. Los pacientes con TB en HZ incurrieron en tasas de reembolso efectivo más elevadas, pero la incidencia del CHE siguió siendo más alta. La reducción de la incidencia del CHE después del NCMS no presentó ninguna diferencia estadísticamente ($P > 0,05$). La gravedad del CHE se vio levemente aliviada. Los CI después del reembolso fueron todos por debajo de cero y los valores absolutos fueron más altos que los que había antes del reembolso.

Conclusiones: El bajo reembolso para los pacientes con TB podría generar una pesada carga financiera. Los pacientes con TB pobre incurrieron en tasas de CHE altas. Se observó que el NCMS era un factor protector del CHE, pero el impacto fue modesto y la equidad de CHE no mejoró. Las políticas de reembolso del NCMS deben mejorarse en el futuro e incluir una cobertura de atención más completa. Puede ser necesario implementar programas adicionales para extender la cobertura de atención de la TB.

Translated from English version into Spanish by María Emilia Meini, through

TRANSLATORS
WITHOUT BORDERS
